# Supplementary material for: The interactome of intact mitochondria by cross-linking mass spectrometry provides evidence for coexisting respiratory supercomplexes
Source: Mol Cell Proteomics. 2017 Dec 8;17(2):216–32. doi: 10.1074/mcp.RA117.000470 (PMC5795388; doi:10.1074/mcp.RA117.000470)
Supplement: Supplemental Data [file supp_RA117.000470_133922_0_supp_23978_fzffwf.zip › spectra_annotation/mito_DR_spectra_annotation/288-1-9-1-2-1.pdf]

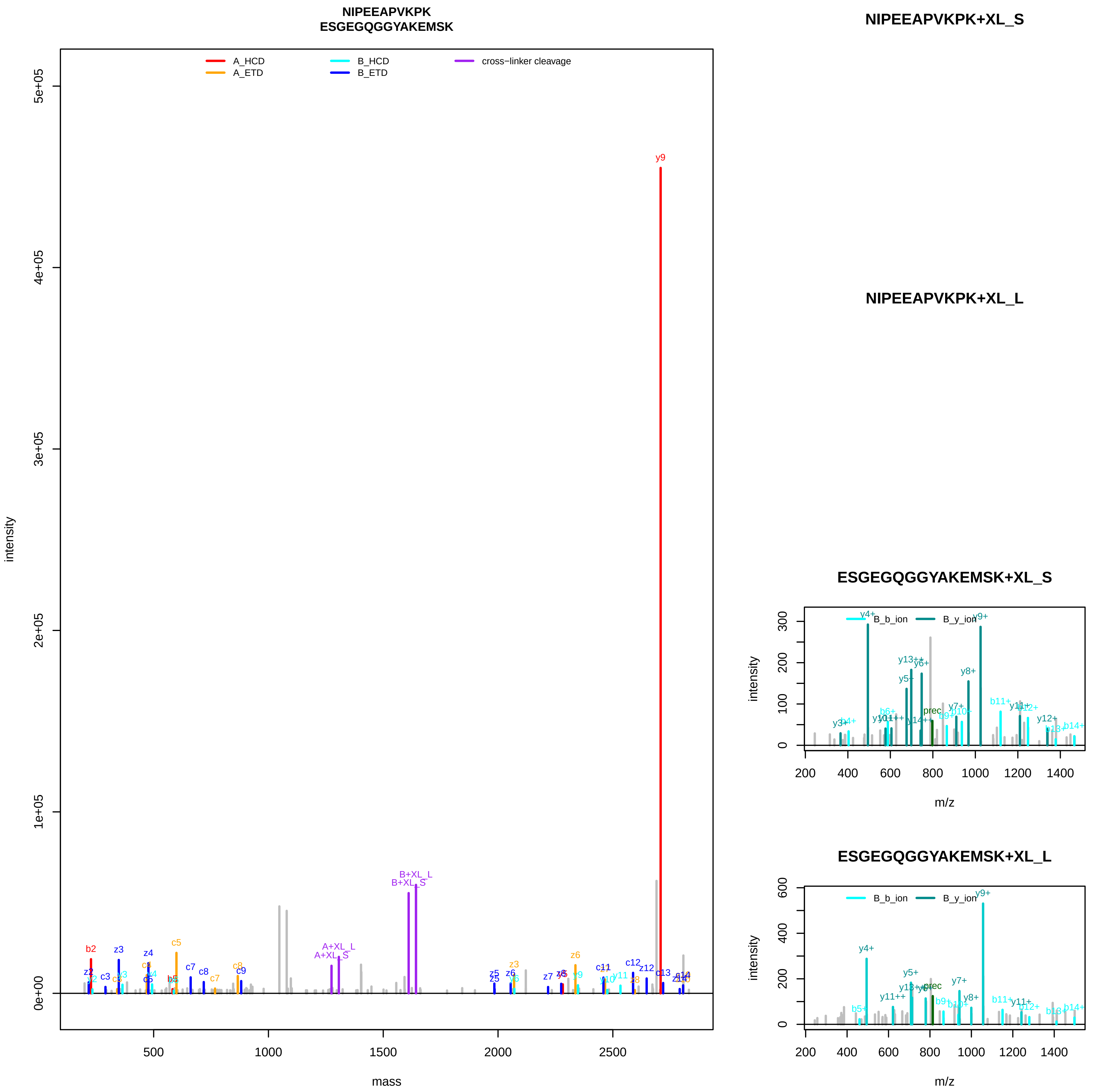
NIPEEAPVKPK
ESGEGQGGYAKEMSK
A_HCD
A_ETD
B_HCD
B_ETD
cross−linker cleavage
intensity
mass
0e+00
1e+05
2e+05
3e+05
4e+05
5e+05
500
1000
1500
2000
2500
y9
B+XL_L
B+XL_S
A+XL_L
A+XL_S
b2
z2
c3
z3
z4
c5
c7
c8
c9
z5
z6
z3
z7
c11
c12
z12
c13
c14
NIPEEAPVKPK+XL_S
NIPEEAPVKPK+XL_L
ESGEGQGGYAKEMSK+XL_S
B_b_ion
B_y_ion
y4+
y9+
y13++
y6+
y5+
y8+
y7+
prec
b11+
y11+
b12+
y12+
b13+
b14+
y3+
b4+
b6+
b9+
b10+
intensity
m/z
0
100
200
300
200
400
600
800
1000
1200
1400
ESGEGQGGYAKEMSK+XL_L
B_b_ion
B_y_ion
y9+
y4+
y5+
y7+
y8+
y11++
b5+
b9+
b10+
b11+
y11+
b12+
b13+
b14+
intensity
m/z
0
200
400
600
200
400
600
800
1000
1200
1400
